# Aptasensors versus immunosensors—Which will prevail?

**DOI:** 10.1002/elsc.202100148

**Published:** 2022-01-13

**Authors:** Sofia Arshavsky‐Graham, Christopher Heuer, Xin Jiang, Ester Segal

**Affiliations:** ^1^ Faculty of Biotechnology and Food Engineering Technion ‐ Israel Institute of Technology Haifa Israel; ^2^ Institute of Technical Chemistry Leibniz University Hannover Hannover Germany; ^3^ Russell Berrie Nanotechnology Institute Technion ‐ Israel Institute of Technology Haifa Israel

**Keywords:** antibody, antibody‐aptamer hybrid, aptamer, biosensors, capture probes

## Abstract

Since the invention of the first biosensors 70 years ago, they have turned into valuable and versatile tools for various applications, ranging from disease diagnosis to environmental monitoring. Traditionally, antibodies have been employed as the capture probes in most biosensors, owing to their innate ability to bind their target with high affinity and specificity, and are still considered as the gold standard. Yet, the resulting immunosensors often suffer from considerable limitations, which are mainly ascribed to the antibody size, conjugation chemistry, stability, and costs. Over the past decade, aptamers have emerged as promising alternative capture probes presenting some advantages over existing constraints of immunosensors, as well as new biosensing concepts. Herein, we review the employment of antibodies and aptamers as capture probes in biosensing platforms, addressing the main aspects of biosensor design and mechanism. We also aim to compare both capture probe classes from theoretical and experimental perspectives. Yet, we highlight that such comparisons are not straightforward, and these two families of capture probes should not be necessarily perceived as competing but rather as complementary. We, thus, elaborate on their combined use in hybrid biosensing schemes benefiting from the advantages of each biorecognition element.

**Abbreviations**
3D: three‐dimensional
Fab/Fab’: antigen‐binding fragment
HCR: hybridization chain reaction
IgG: immunoglobulin G
LOD: limit of detection
mAB: monoclonal antibody
RCA: rolling circle amplification
scAB: single‐chain antibody
scFv: single‐chain variable fragment
SELEX: systematic evolution of ligands by exponential enrichment

## 1 INTRODUCTION

The prominent technological advancements over the past two decades have accelerated the development of biosensors, which aim to replace traditional laborious and time‐consuming analytical methods and enable rapid, sensitive, and simple analyte detection for early diagnosis of diseases, food contamination, and environmental monitoring. This immense progress is evidenced during the global battle against COVID‐19, where rapid point‐of‐care (POC) biosensors are already widely used [1]. Biosensors are analytical devices in which a biological capture probe is used to specifically recognize a target analyte, which is then converted by a transducer into a measurable signal [2]. The proper choice of both elements, as well as their integration, is crucial in achieving sensitive and selective analyte detection.

Antibodies have been the most widely used capture probes in biosensors, as they have been naturally evolved to bind their target analyte with high affinity and specificity and are considered as the “gold standard.” Yet, antibody technology has been suffering from limitations related to its costs, structure, and the unavailability for various important analytes, leading to the search for alternative capture probes [3]. In 1990, three different studies reported the identification of RNA binding ligands for different targets, by a similar selection process [4, 5, 6]. This process was termed Systematic Evolution of Ligands by Exponential Enrichment (SELEX), and since then enabled the identification of numerous nucleic acids, termed aptamers, that specifically bind various target molecules. Aptamers, also termed “chemical antibodies,” are single‐stranded oligonucleotides (or peptides), which bind their target through their specific three‐dimensional (3D) conformation. Their chemical structure, size, and synthetic production have been promising for overcoming some of the disadvantages of antibodies and stimulated the research in their integration as capture probes in biosensing platforms.

This review intends to provide a comprehensive comparison of aptamers and antibodies as capture probes in biosensing platforms. We survey the advantages and recent developments of each probe class as well as the different detection concepts and schemes they provide. We also focus on aspects to be considered in the immobilization or integration of these capture probes with the transducer element of the biosensor. We compare both capture probes and review studies that presented their experimental comparison in similar settings. Finally, we describe biosensors combining both capture probes in a hybrid approach and the capabilities of such systems.

## 2 IMMUNOSENSORS

### 2.1 Antibodies and their derivatives as capture probes in immunosensors

**FIGURE 1:**
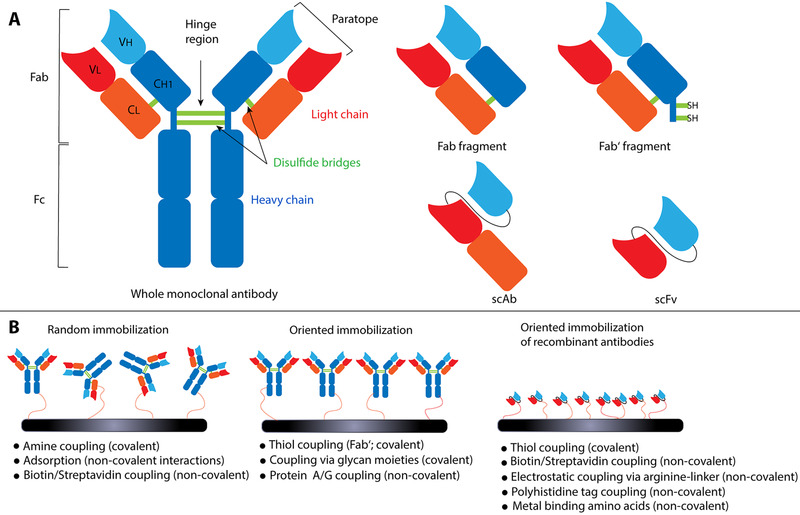
Antibodies as capture probes in immunosensors. (A) Schematic overview detailing the structure of whole antibodies and their fragments such as Fab, Fab’, scAb and scFv. (B) Strategies for immobilization of antibodies and their derivatives onto biosensor surfaces. These strategies include various covalent and non‐covalent approaches for random and oriented biosensor functionalization

Antibodies are presumably the most important and prominent class of biorecognition elements [7, 8]. In vivo, these proteins play a crucial role in the adaptive immune system of vertebrates targeting antigens, such as pathogenic microorganisms, with high affinity and specificity [7, 9]. Antibodies consist of two moieties (each containing a light and a heavy chain) bound together by disulfide bonds; this region is referred to as the hinge region (see Figure 1A) [10, 11]. The top of the heavy and light chains variable regions (V_H_ and V_L_, respectively) form the paratope directed against a specific structure (epitope) on the antigen [10]. By binding these epitopes, antibodies can neutralize pathogens or mark them to trigger other immune responses [9, 12]. In nature, antibodies produced during the immune response against a specific target are polyclonal, descending from different B cell clones, and they recognize and bind different epitopes on the same antigen [13]. The development and production of monoclonal antibodies (mAb), descending from identical B cells and as such targeting a single antigen's epitope, have boosted the usage of antibody‐based recognition elements for diagnostic applications [9, 13]. These antibodies are widely used today in numerous bioanalytical assays, including the common enzyme‐linked immunosorbent assay (ELISA) [14]. Importantly, antibodies as well as their derivatives are widely employed as capture probes in biosensing schemes, and the resulting biosensors are correctly referred to as immunosensors [9, 15].

Various antibody‐based biorecognition elements, such as whole mAbs but also derivatives like antigen‐binding fragments (Fab, Fab’), single‐chain variable fragments (scFv), or single‐chain antibodies (scAb), are available as capture probes for immunosensor applications [9, 10]. Figure 1A depicts a schematic overview of different antibody‐based biorecognition elements. Fab fragments consist of four domains (V_L_, C_L_, C_H1_, V_H_) and Fab’ fragments feature an additional thiol group originating from the disulfide bridges of the hinge region in whole antibodies [10]. In immunosensors, Fab’ fragments are preferred over Fab fragments as the C‐terminal thiol allows for simple and oriented immobilization on the sensor/transducer surface [10, 16]. Also, their production by protease‐mediated cleavage of whole mAbs is straightforward and relatively cheap [16]. Examples of non‐naturally derived antibody fragments that can be obtained by recombinant protein production using microbial expression systems are scFvs and scAbs. In these fragments, the V_H_ and V_L_ domains are connected by a genetically engineered internal protein chain linker with a typical size of 15–30 amino acids; scAbs feature an additional C_L_ domain to improve the stability and expression of these antibody derivatives (Figure 1A) [9, 10, 17]. These recombinant fragments allow for high customizability as genetic engineering enables incorporating desired protein sequences such as C‐terminal thiols or polyhistidine tags [9, 18]. These approaches, along with the maturation of antibodies manufacturing and processing technologies, including their large‐scale recombinant production in various host systems, have reduced their costs as well as the variability between batches [19, 20].

### 2.2 Immunosensor design considerations: Capture probe size and immobilization routes

Critical aspects affecting the immunosensor performance are the capture probe size and its immobilization onto the sensor surface. Antibody fragments, such as Fab’ (∼50 kDa) [21], scAb (∼40 kDa) [22] and scFv (∼30 kDa) [23], are significantly smaller than whole mAbs (∼150 kDa) [23] and as such they can be more densely immobilized onto an immunosensor surface, potentially allowing for improved sensitivity and lower limits of detection (LOD) [10].

The antibody immobilization onto the sensor surface is crucial for maintaining its proper conformation and correct orientation to allow optimal interaction with the target analyte [9, 24]. Most common immobilization routes, including adsorption and amine coupling, result in random orientation, as schematically illustrated in Figure 1B, limiting the binding site's accessibility [8]. Thus, it is highly desirable to direct a tail‐on antibody orientation on the surface, with the Fc region coupled to the surface and the antigen binding site towards the target solution (Figure 1B) [25]. Covalent and oriented immobilization can be achieved by coupling via glycan moieties or using Fab’ fragment's thiol groups; the latter can be linked to maleimide‐modified surfaces or directly to gold surfaces [10, 16, 26]. Oriented affinity‐based immobilization relies on the usage of protein A (produced by *Staphylococcus aureus*) or protein G (from *Streptococcus* species), which exhibit high affinity towards the Fc region of antibodies, and can be used as intermediate proteins between the biosensor surface and the antibody [24, 27]. While protein A does not recognize all immunoglobulin G (IgG) subclasses, protein G is less specific and features serum albumin binding sites [28, 29]; these characteristics should also be considered in the immunosensor design. Recombinant antibody‐fragments such as scAbs and scFvs yield the opportunity to genetically engineer protein sequences and precisely define the positions used for immobilization by inserting specific amino acids and functional groups; therefore, enabling specific and oriented biosensors functionalization [9, 10]. For example, C‐terminal thiol groups or streptavidin binding peptides have been fused to recombinant antibodies [18, 30]. C‐terminal Avi‐Tag, which is recognized in vivo by the BirA enzyme and biotinylated, provided the means for oriented immobilization on streptavidin‐coated surfaces, resulting in >200‐fold analyte binding improvement compared to random immobilization [31]. Also, the peptide linker used in scFv and scAb fragments can be modified with polyhistidine tags and other metal‐binding amino acids or positively charged arginines for electrostatic coupling to obtain a controlled antibody immobilization [9, 32, 33]. For example, scFv constructs in which a polycationic arginine peptide was used for immobilization on negatively charged surfaces improved by 42‐fold the detection of rabbit IgG (as a target) in comparison to Fab antibodies [32].

Immunosensors are mostly integrated in conventional sensing formats such as direct, sandwich, and competitive assays. Direct immunosensors are the most straightforward type of antibody‐based biosensors, where the target analyte is introduced to the sensor surface, which is functionalized with the desired capture probe, and upon analyte binding, the transducer converts this event into a measurable signal [34]. Sandwich immunosensors employ two antibodies and typically feature the highest sensitivity and specificity. In this sensor format, the target analyte is “sandwiched” between an antibody immobilized onto the sensor surface and a second antibody that is subsequently introduced [35, 36]. Therefore, the target analyte is required to have epitopes for both antibodies, and as such, the detection of small target molecules can be challenging [37]. In competitive immunosensor formats, which are considered advantageous for small molecule detection, the target analyte derived from a clinical or environmental sample and an artificially labeled analyte compete for the antigen binding site of the immobilized antibody capture probe. As the detectable signal is typically measured from the artificially labeled analyte, it decreases with an increased concentration of the “real” target analyte [36, 38].

## 3 APTASENSORS

**FIGURE 2:**
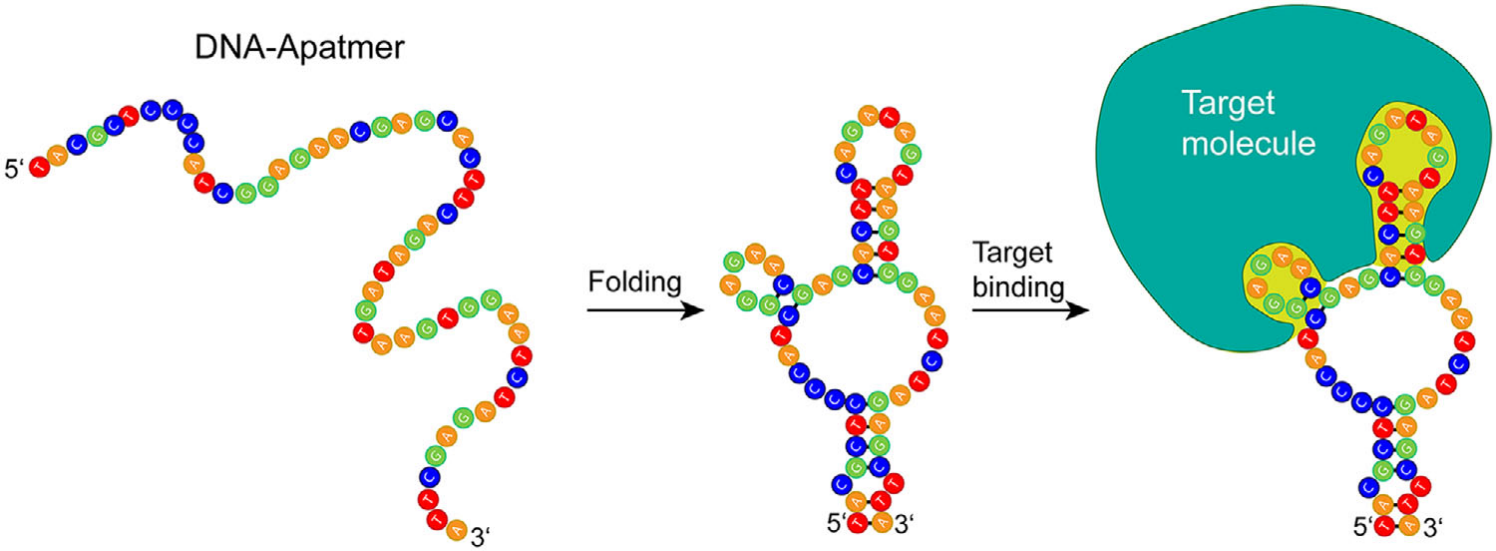
Illustration of the 3D folding of aptamers and their binding to their target analyte

Aptamers are single‐stranded oligonucleotides (or peptides), usually 20–100 bases long, which fold into a 3D structure enabling the specific binding of their target analyte. The binding between an aptamer and its target is non‐covalent and governed by various forces, such as hydrogen bonds and van der Waals forces, forming 3D configurations, such as stem, loop, hairpin, or G‐quadruplex structures [39], which are schematically illustrated in Figure 2. The binding is characterized by a high affinity, with dissociation constant values down to picomolar, and a high specificity. In fact, aptamers can discriminate between subtle structural differences, such as enantiomeric configuration or the presence of a methyl group [40, 41, 42, 43]. Aptamers are selected using the SELEX process in which very large combinatorial libraries of oligonucleotides are screened in an iterative process of in vitro selection and amplification for their affinity to a specific analyte [44, 45, 46]. The libraries, containing 10^13^–10^18^ DNA or RNA sequences, are first incubated with the target of interest in a buffer of choice and those which bind the target are then separated from the rest by physical or chemical methods and eluted, followed by their amplification to generate a new pool for the next selection cycle. The final enriched library, comprised of the sequences presenting the strongest binding to the target, is cloned and sequenced, followed by the investigation of individual sequences for their target affinity. The advantages of this process are that (1) the selectivity can be increased by performing a counter selection for removal of aptamers that bind to structures similar to that of the target; (2) it can be performed in a medium of choice and potentially select aptamers that are active in non‐physiological conditions; (3) it can be accomplished without knowing the specific target or antigen, such as selection of aptamers binding a cell or a tissue in cell‐SELEX [47, 48, 49, 50] or in vivo SELEX [51], respectively; and (4) the process enables the selection of aptamers for non‐immunogenic molecules, toxic molecules or pathogens. Typically, the selection process requires 6–20 cycles and takes weeks to months to complete [52, 53]; yet once completed, the aptamers can be produced in sizeable quantities, high purity, and constant quality by standard chemical synthesis. Furthermore, SELEX techniques have been constantly advancing, becoming more efficient, straightforward and of routine practice, by methods such as Graphene oxide SELEX [54], magnetic bead‐based SELEX [55, 56], capillary electrophoresis SELEX [57, 58], fluorescence‐activated cell sorting SELEX [59], one‐round SELEX [60, 61, 62, 63] and capture SELEX [64]. Indeed, aptamers have been selected to target a broad range of analytes, including metal ions [65, 66, 67], small organic molecules [68, 69, 70], proteins [71, 72], whole cells [73, 74] and microorganisms [75, 76]. Aptamers have been selected for diagnostic applications, targeting various disease biomarkers [77, 78, 79, 80], as well as for therapeutic applications, for the discovery of new biomarkers, for cancer imaging, for targeted delivery, and as therapeutic agents [81, 82, 83, 84, 85, 86].

When compared to antibodies, aptamers present several significant advantages [52, 53, 87, 88]. First, as aptamers are mostly produced in an animal‐free synthetic approach, their production is simpler, less expensive, more robust, and scalable. In contrast, the purification and processing of antibodies can be tedious, resulting in variability between batches. Second, the chemical synthesis of aptamers also enables flexibility in tuning the aptamer sequence in a specific manner to improve its affinity to the target molecule [89]. For instance, nucleobases can be modified to tighten the binding with the target, such as the Slow Off‐rate Modified Aptamers (SOMAmer), which incorporate modified dUTPs at the 5′‐position that participate in the interaction with the target, resulting in superior affinity and nuclease resistance [90]. Their backbone can also be replaced to form artificial DNA analogs, such as the peptide nucleic acids, in which the phosphate‐ribose is replaced with an amide backbone, presenting an electroneutral structure, improved affinity, and chemical stability [91]. Third, aptamers are smaller, facilitating their immobilization on surfaces, providing more target binding sites while minimizing steric hindrance effects. Consequently, the aptamer‐bound target is located closer to the transducing surface, which can be advantageous for increasing the biosensing signal [92], for example, in surface Plasmon resonance (SPR) biosensors [93, 94]. Aptamers are also advantageous for the detection of small molecules and ions when compared to antibodies [95], owing to their capability to undergo significant conformational changes upon target binding, leading to higher signal and sensitivity. Finally, in terms of stability, antibodies are usually limited to physiological conditions and are prone to irreversible denaturation, whereas aptamers (specifically DNA‐based) exhibit superior chemical stability, allowing to function in harsh conditions (depending on their selection process), and can undergo reversible folding and unfolding. It should be noted that the latter advantage clearly depends upon the desired application and designated environmental conditions of the specific biosensor. The number of aptamers that are selected in real biological fluids is limited [50, 96], while the majority is selected under defined buffer conditions. As such, in cases in which the biosensor operates in biological fluids and under physiological conditions, antibodies are often superior as unmodified DNA and RNA aptamers are prone to nuclease degradation [10]. The aptamer's reversible folding also allows to easily elute the bound target from the aptamer for its regeneration for repetitive usage; this can be performed by simple heat treatment or by conditions predefined during their selection process, whereas for antibodies, the elution conditions need to be tediously probed to avoid irreversible denaturation [97]. These features make aptamers a promising alternative for antibodies and attractive capture probes for biosensor design.

### 3.1 Aptasensor design considerations

Construction of an aptasensor requires the conjugation of the aptamer to a transducing surface or a functional material. This can be achieved via non‐covalent interactions, which is the most straightforward and simple strategy. For example, the DNA bases can form coordination interactions with gold surfaces [98] or hydrogen bonds and π‐π stacking with material sfeaturing aromatic ring moieties, such as graphene oxides [99]. Yet, such non‐covalent interactions are less stable, and the control over the orientation and uniformity of the immobilization is challenging. Alternatively, modification of the aptamers with various functional groups at specific locations (i.e., the 5′‐end, 3′‐end and/or internally within the sequence), already during their synthesis, enables their facile covalent conjugation at desired orientations [86, 100]. In contrast, immobilization of antibodies, which is most often limited to protein natural functional groups, results in an unknown immobilization configuration, non‐uniformity, and undesired complexation, while the control over orientation is more complicated.

Besides the choice of the chemical conjugation route, aptamer immobilization should also be tailored in terms of the surface coverage density and orientation to achieve the proper functional folding of the aptamers [100, 101, 102]. Proper aptamer density on a surface is crucial for achieving sufficient binding sites for the target, while minimizing steric hindrance and electrostatic repulsion between neighboring aptamers. As such, aptamer immobilization onto nanomaterials and nanostructured surfaces, which are more prone to steric crowding effects, should be carefully designed [97, 100, 103‐105]. Furthermore, excessive density may also affect the analyte mass transfer rate, as was demonstrated for surface‐based biosensors [106, 107]. Thus, the concentration of the aptamer used for immobilization should be carefully optimized to yield a surface density that allows the best biosensing performance [106, 108‐112]. Besides their surface density, the aptamer orientation on a surface (binding via the 5′ or 3′ terminus), may affect its proper folding and resulting functionality. This is dictated by the location of the terminus within the binding region of the aptamer and its involvement in the folding. Thus, both immobilization directions should be screened and compared for best results [100, 102, 103, 111, 113]. Moreover, when surface‐based aptasensors are considered, incorporation of a spacer to extend the binding region of the aptamer from the surface and minimize steric hindrance effects may also improve the aptamer functionality. The spacer can be a polyethylene glycol moiety fused with the aptamer sequence or on the surface, or by direct sequence elongation, such as the introduction of several thymine bases [102, 103, 109, 111, 113‐115]. The last aspect to be considered is preventing undesired electrostatic interactions between the negatively‐charged aptamers and positively‐charged surfaces, which may impede the aptamer's folding; this can be achieved via surface modification or passivation strategies [100, 102].

### 3.2 Different concepts of aptasensing

**FIGURE 3:**
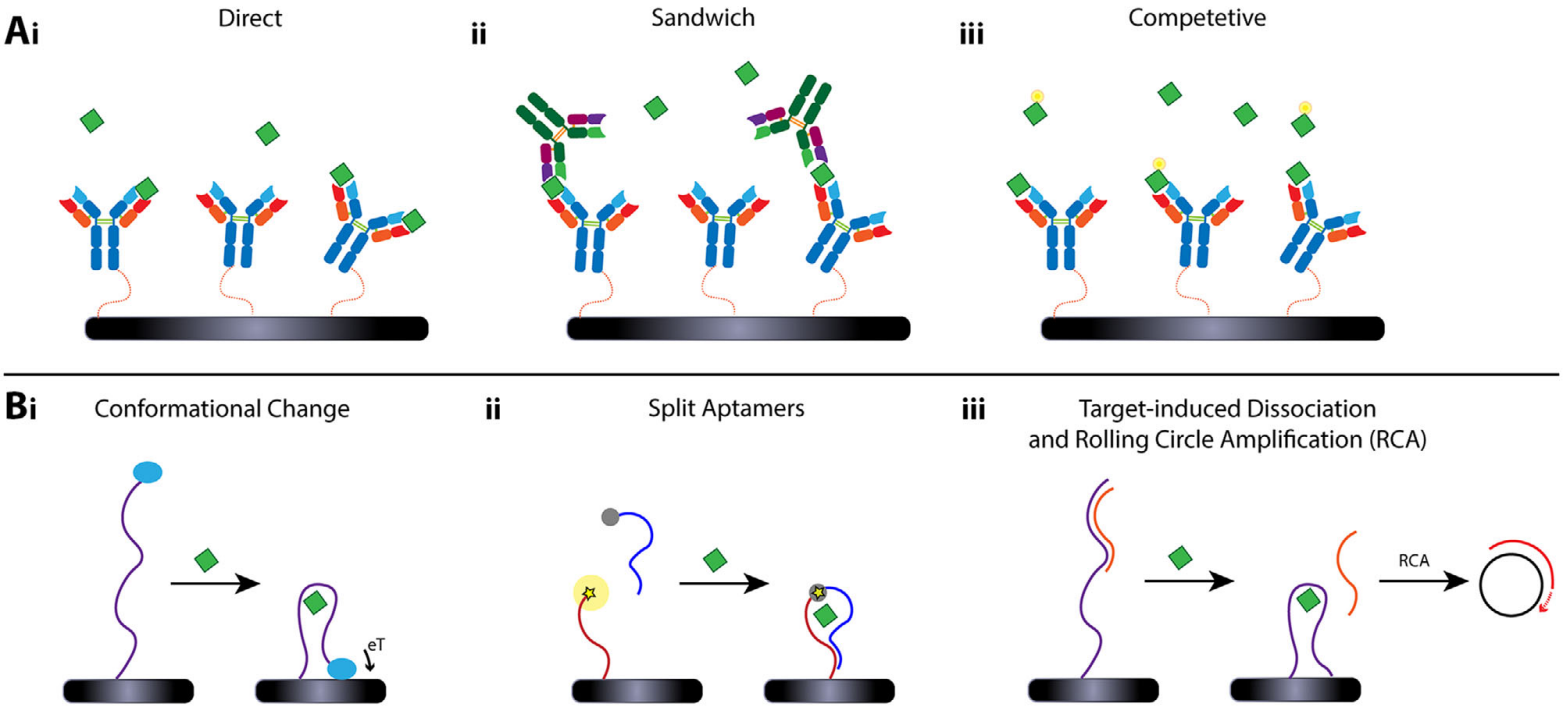
Immunosensor and aptasensor biosensing concepts. (A) Immunosensors are often used in conventional sensing formats. (i) In direct assays, the target analyte binds to the capture probe, which is immobilized onto the sensor surface, while in sandwich assays (ii) a secondary antibody is used to increase the selectivity and sensitivity of the assay. (iii) In competitive assays, an artificially‐labeled analyte and the “real” target analyte compete for the capture probe binding sites. (B) While aptamers can also be used in such conventional sensing schemes, more elaborated concepts have been developed. (i) Target binding induces conformational changes of the aptamer, which is labeled with a redox‐active molecule, altering electron transfer (eT) and thus the measurable signal. (ii) The binding of split aptamers upon analyte binding causes a detectable signal. For instance, one aptamer that is labeled with a quencher molecule represses the fluorescence of the second fluorescently labeled aptamer. (iii) Target binding can cause a complementary DNA strand to dissociate that is subsequently used as a primer in an RCA reaction to increase the sensitivity

While immunosensors have been mainly used in traditional forms of direct, sandwich or competitive target detection (see Figure 3A), the ability of DNA and RNA aptamers to hybridize by Watson‐Crick base pairing with complementary strands, as well as their conformational mechanism of target binding, allow for sophisticated biosensing schemes with extended capabilities and analytical performance [89, 116].

Aptamers can be re‐engineered to undergo binding‐induced conformational changes, which improve both the sensitivity and the selectivity of the biosensor. The latter is highly relevant for detecting small molecules, such as toxins, pesticides, drugs, antibiotics, and hormones, as their detection by conventional antibody‐based approaches is limited [117, 118, 119, 120]. The binding‐induced conformational changes of aptamers and their rapid response enabled the development of biosensors for continuous real‐time detection, demonstrated in the seminal work of Plaxco and coworkers [121, 122, 123, 124, 125]. They developed electrochemical aptamer‐based sensors in which the aptamers are immobilized onto an electrode and are labeled with a redox probe, see Figure 3B‐i. Target binding to the aptamer alters its conformation, which in turn affects the proximity of the redox tag to the electrode, and the resulting measured current in “signal‐off” or “signal‐on” modes. The rapid on‐ and off‐rates of target binding in such aptasensors allow them to perform directly in flowing biological fluids and in awake animals [126, 127].

A related aptasensing concept is the use of split aptamers [125, 128, 129], see schematic illustration in Figure 3B‐ii, where the aptamer sequence is split into two (or more) fragments, which assemble only upon the target binding. Each of the aptamer fragments can be functionalized with labels or amplification moieties to enable signal generation upon binding and its amplification [130, 131, 132]. The main challenge in this sensing mechanism is the stability of the formed split aptamer‐target complex. For this matter, the split aptamers fragments can be “locked” in the correct structure upon the target binding using chemical modifications [89].

One of the unique characteristics of aptamers is their ability for exponential self‐amplification, which can be harnessed for improving the sensitivity of biosensors isothermally, without thermal cycling. In most cases, the amplification technique is target‐triggered and can be coupled with aptamers in various manners [133, 134, 135, 136, 137, 138]. The aptamer sequence and the amplification‐active component can be conjugated to create a single probe with dual functionality, where upon target binding, the aptamer can induce or inhibit the process by conformation change or by releasing a complementary strand that initiates the process. Such amplification may substantially enhance the collected signal and improve the LOD by orders of magnitude [89].Common amplification methods include rolling circle amplification (RCA) [133, 134], hybridization chain reaction (HCR) [135, 136, 137], loop‐mediated isothermal amplification (LAMP) [139, 140] and DNA walker [141]. Figure 3B‐iii schematically depicts the concept of RCA, where a DNA polymerase extends a primer based on a circular template. The latter can be a complementary DNA strand that dissociates from the aptamer upon target binding, while the primer remains bound to the aptamer when no target binding occurs, and the RCA reaction cannot be initiated [142].

## 4 EXPERIMENTAL COMPARISON OF APTASENSORS AND IMMUNOSENSORS

Over the past 2 decades, numerous aptamer‐based biosensors have been reported, with many studies suggesting their superiority over immunosensors [52, 143]. Yet only several studies have experimentally compared between immunosensors and aptasensors under equivalent conditions, presenting inconsistent results [97, 113, 144‐146]. The biosensing performance, in terms of selectivity and sensitivity, has been shown to be similar for aptasensors and immunosensors, for several types of transducers, such as quartz crystal‐based [113], nanogap impedance‐based [145], nano‐modified screen‐printed electrodes [144], as well as for porous silicon thin films [97]. Yet, a critical factor affecting this performance is the immobilization route of the different capture probes [10, 97, 147]. In our recent report, we have compared the performance of optical porous silicon‐based biosensors for targeting a family of proteins, where aptamers and antibodies are employed as capture probes. We demonstrated that only upon oriented immobilization of the antibodies on the surface, comparable apparent binding rate, dynamic detection range, sensitivity, and selectivity are obtained for the immunosensor and the aptasensor. Several studies reported superior analytical performance of aptasensors in comparison to their corresponding immunosensors [146, 148]. Yet, it should be noted that in these reports, the antibodies were immobilized in an unfavorable random orientation, which impedes their antigen binding capacity. Clearly, the overall biosensor performance is also dependent on the transducer element, which may be differently affected by the capture probe molecule characteristics. For example, certain transducers will benefit more from the aptamer's small size and its closer proximity to the surface [92, 149].

The main advantages of aptamers over antibodies are therefore their enhanced stability, versatile design, and ability to be regenerated for reusable biosensors [97, 113, 144]. Aptasensors' shelf life is significantly longer compared to immunosensors; it was demonstrated that aptasensors can be stored for several weeks without sensitivity loss [113], as well as in dry conditions [97]. Contrary, immunosensors storage is usually limited to several days, under refrigeration and wet conditions [97]. Regeneration of aptasensors can be easily achieved without hampering their activity, allowing for the construction of reusable biosensors [97, 113, 117]. For immunosensors, regeneration is more challenging, and the conditions must be probed tediously. Even if the bound target is released from the immunosensor, a significant performance loss is commonly observed due to irreversible damage to the antibodies [97, 113]. Thus, the regeneration of immunosensors is highly dependent on the specific antibody and its durability. In contrast to antibodies, aptamer's small size allows their immobilization in a dense receptor layer on the biosensor surface. This contributes to extending the biosensor's detection range, increasing the biosensor response and the target binding capacity [97, 113].

## 5 HYBRID SYSTEMS

Hybrid systems, in which both capture probes (i.e., antibody and aptamer) are combined within the same biosensing platform, have been gaining considerable interest in recent years [150, 151]. The most common hybrid systems rely on a sandwich configuration, where the target analyte is bound by a first capture probe, followed by the introduction of a secondary reporter capture probe which amplifies the signal or includes labels. While sandwich antibody‐antibody assays are prevalent, the substitution of one of them with an aptamer capture probe was found to be highly advantageous in numerous biosensing platforms [151, 152, 153, 154, 155, 156, 157]. Furthermore, as such sandwich assays often require a dual‐site recognition of the target, aptamers and antibodies complement each other in this manner [150]. Sandwich aptamer‐antibody biosensors have been developed for different targets, such as proteins [152, 154, 155, 158‐162], antibiotics [163, 164], bacteria [165, 166] and fungi [167, 168] in various biosensing platforms. These have been recently summarized in an excellent review by Jarczewska and Malinowska [150]. Thus, in this section, we only briefly survey the main aspects of such biosensors with a few recent examples.

**FIGURE 4:**
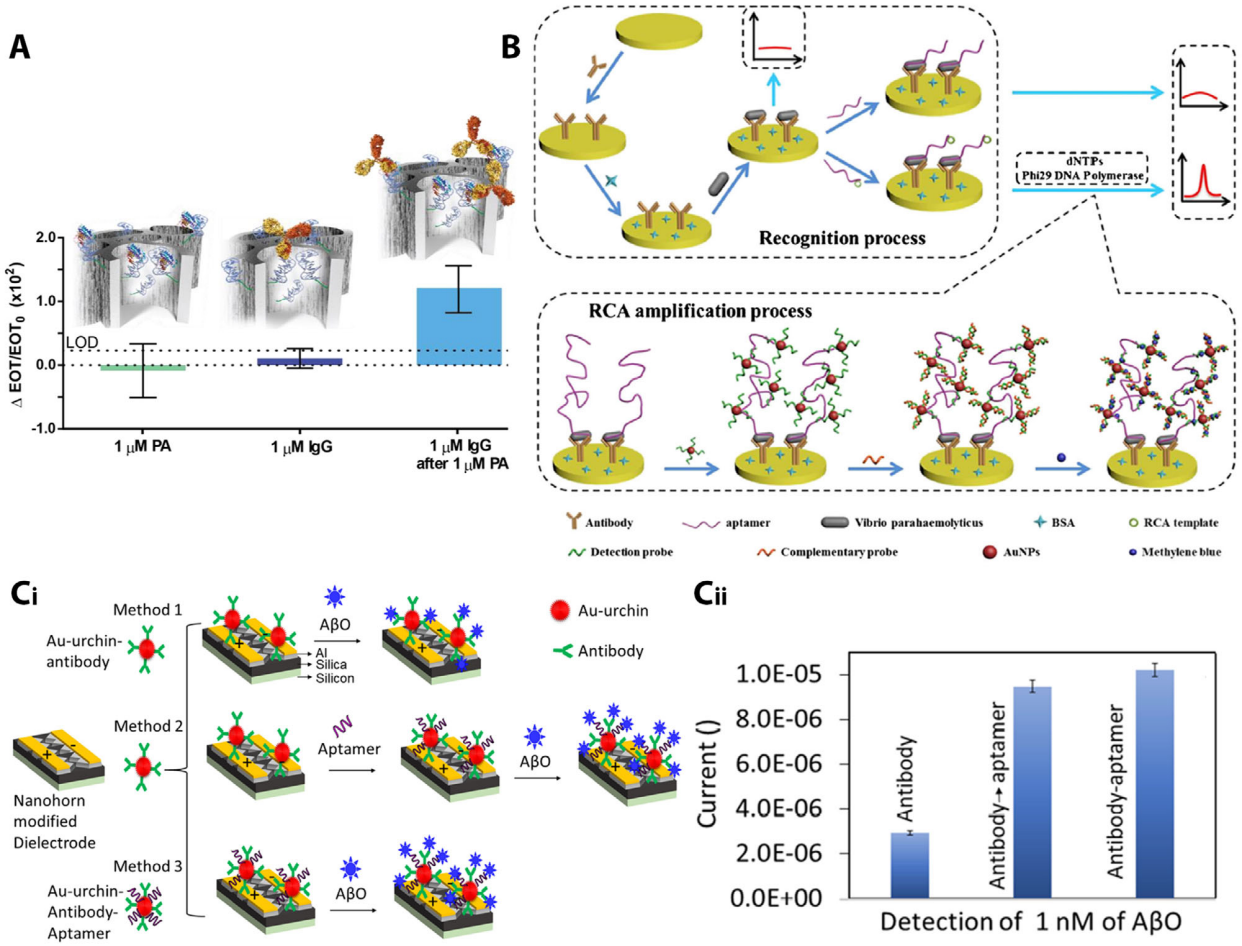
Hybrid biosensors employing aptamers and antibodies as capture probes. (A) Sensitivity enhancement by application of an antibody as a secondary capture probe for detection of protein A with an aptamer‐functionalized porous silicon biosensor. Reproduced with permission from [169]. Copyright 2017, Elsevier B.V. (B) Schematic illustration of the RCA based hetero‐sandwich electrochemical biosensor for ultrasensitive detection of *Vibrio parahaemolyticus*. Reproduced with permission from [166]. Copyright 2017, Springer Nature. (C‐i) Schematic illustration of an aptamer‐antibody dual probe system for amyloid‐beta (AβO) determination, based on a nanohorn‐modified dielectrode. The dual probe system was compared to a single antibody probe (top panel), as well as to a single step (bottom panel) and a two‐step (middle panel) immobilization routes of both capture probes. (C‐ii) Comparison of biosensing signal for detection of amyloid‐beta with the different platforms, presenting improved performance for the dual probe scheme. Adapted with permission from [176]. Copyright 2021, the authors. Published under Attribution‐NonCommercial 3.0 Unported (CC BY‐NC 3.0)

Many of the sandwich assays use the aptamer as the primary capture probe, while the antibody is used as the reporter probe owing to its higher molecular weight and larger size, which can induce a higher biosensing signal amplification [150]. For example, Urmann et al. have designed an aptamer‐antibody hybrid system for improving the sensitivity of porous silicon‐based optical aptasensor for targeting protein A, a biomarker for *Staphylococcus aureus* [169]. While low concentrations of the protein were not detected by the aptasensor (Figure 4A), the subsequent introduction of an (unlabeled) IgG probe, which binds to the aptamer‐immobilized target, improved the LOD of the aptasensor by three‐fold. Alternatively, the aptamer can be employed as the reporter probe [153, 166, 168, 170], where the oligonucleotide nature of the aptamers allows for distinctive amplification strategies (as described in Section 3.2). A popular biosensing configuration involves immobilization of a capture antibody on the transducing element (such as an electrode); the aptamer binds to the captured analyte and induces a cascade of subsequent reactions to allow sensitive detection [150]. Figure 4B presents the concept of such a hybrid amperometric biosensor for detection of the foodborne pathogen *Vibrio parahaemolyticus* [166]. The target pathogen is first captured by a specific antibody, which is immobilized onto a gold electrode, followed by the subsequent introduction of an aptamer probe, which includes a template for RCA. Upon several labeling steps of the amplification products of the latter process (see Figure 4B, lower panel), highly sensitive detection of the pathogen is achieved (LOD of 2 cfu mL^‐1^). Another elegant antibody‐aptamer sandwich assay in which RCA is employed was demonstrated for a model protein detection [171], where an oligonucleotide probe comprising of a thrombin‐binding aptamer and a primer sequence for RCA was utilized. The RCA reaction generated a long sequence of aptamers, which could bind many thrombin molecules, catalyzing the substrate's cleavage into detectable products, resulting in sensitive detection in the picomolar range. Antibody‐aptamer hybrid biosensors employing HCR amplification were also reported [151, 172, 173]. For example, Zhang et al. have applied this approach for fluorescence‐assisted detection of tumor‐related exosomes [162], where the exosomes were sandwiched between CD63 antibody‐functionalized magnetic nanoparticles and oligonucleotides, comprising of a PDL‐1 (Programmed death‐ligand 1) targeting aptamer and an initiator of HCR. In such hybrid systems, the antibody and the aptamer capture probes can be also employed simultaneously as demonstrated by Ma et al. [174] for the detection of mucin 1, a cancer protein biomarker. Fluorescent carbon dots (CDs) were functionalized with anti‐mucin 1 antibody or aptamer; exposure of their mixture to the target protein forms a sandwich complex, which induces aggregation of CDs and subsequent quenching of the fluorescence signal. The latter was correlated to mucin 1 concentration, resulting in a LOD of 2 nM, and selective performance in diluted serum samples.

Several works have characterized and compared the performance of aptasensors and hybrid biosensors for specific targets. Bin et al. [175] compared the analytical performance of an SPR biosensor for C‐reactive protein detection in several configurations. They demonstrated that hybrid aptamer‐antibody biosensors outperform the aptasensor in terms of LOD (from 1 to 0.1 nM in a standard sandwich format and to 10 pM in a gold nanoparticle‐enhanced sandwich measurement). Jian et al. [148] investigated the performance of carbon nanotube‐modified electrode biosensors for targeting the clotting factor IX using specific aptamer and antibody capture probes, as well as their hybrid sandwich formats (aptamer‐antibody and antibody‐aptamer). Interestingly, for the aptamer‐immobilized surface, the sandwich format with an antibody did not result in LOD improvement, while for the antibody‐immobilized surface, the sandwich format with the aptamer resulted in a ten‐fold improvement in the LOD. Marta et al. investigated both sandwich formats for electrochemical biosensor for the detection of the Human Epidermal growth factor Receptor 2 (HER2) biomarker [170]. Although the aptamer immobilization on the gold electrode was simpler, its use as the capture element and the antibody as the reporter did not result in a reliable signal. This was attributed to the separation of the antibody‐HER2 complex from the surface due to their stronger interaction. In contrast, improved performance was attained when immobilizing the antibodies on the electrode and using methylene blue (MB)‐labeled aptamer as the reporter probe. Yet, it should be noted that sandwich assays do not always result in improved performance compared to direct detection schemes [150].

Recently, a hybrid aptamer‐antibody biosensor that employs both probes in a dual manner has been reported by Qiu et al. [176]. They presented a carbon nanohorn‐modified electrode, functionalized with a dual probe of an aptamer‐antibody‐modified Au urchin for detection of amyloid‐beta, an Alzheimer's disease biomarker; see Figure 4C‐i. The dual probe system was compared to a single antibody probe, as well as to a single step and a two‐step immobilization routes of both capture probes. Both dual probe surfaces resulted in significantly improved performance; however, simultaneous immobilization of both the antibodies and aptamers resulted in higher signals, attributed to more target binding sites (see Figure 4C‐ii).

To summarize, in most cases, aptamer‐antibody (and antibody‐aptamer) hybrid systems have shown to be superior in terms of their analytical performance compared to direct aptasensors or immunosensors. Moreover, antibody‐aptamer constructs provide the possibility to overcome limitations of weak specificity under conditions relevant for the detection of clinical or environmental analytes [177]. Yet, it should be kept in mind that these hybrid assays are often more complex and lengthier as they require multiple steps.

## 6 CONCLUDING REMARKS

The intrinsic advantages of aptamers and their distinctive properties have predestined them as superior capture probes for biosensor design and their use has been increasingly rising. Aptamers have opened the way for overcoming traditional limitations of immunosensors, such as size, stability, variability between batches, cost, and chemical modifications, while presenting new possibilities and concepts for enhancing biosensor sensitivity, selectivity, and response time. Aptamers are especially vital for designing biosensors for detecting small molecule analytes, which has been a challenging task with traditional immunosensors. Yet, both aptamer and antibody performance critically depends on their proper immobilization and integration with the transducing element, which may affect the results of the experimental comparison of both capture probes.

Notwithstanding the advantages of aptamers, antibodies are still the first choice for a capture probe in biosensors. This is mostly ascribed to the extensive usage of antibodies as capture probes for biosensor design for over 70 years and to some extent to the advancements in antibody technology with the introduction of antibody fragments and recently emerging nanobodies [9, 178]. Also, it should be kept in mind that aptamers, being nucleotides, are intrinsically limited in their functional diversity in comparison to proteins. Moreover, as the affinity of different aptamers to their respective target vary considerably (with dissociation constant values in the range of sub‐nanomolar to micromolar [179, 180]), switching from one aptamer to another on a specific biosensor platform is not always straightforward. Therefore, many biosensing studies are conducted on well‐established and characterized aptamer sequences, such as the thrombin aptamer. However, the SELEX process is being constantly improved, and the availability of aptamers is anticipated to increase exponentially. Furthermore, a single capture probe may be replaced by dual capture probe systems with the opportunity to apply the advantages of both capture probes in the same system. This has given rise to the development of hybrid systems, demonstrating promising results, and improved analytical performance with the potential to be tremendously expanded in the near future.

## ACNOWLEDGMENT

This work was funded by the German Research Foundation under the grant SCHE 279/32‐1.

## CONFLICT OF INTEREST

The authors have declared no conflicts of interest.

## DEDICATION

This paper is dedicated to Prof. Thomas Scheper, a pioneer biotechnologist, an inspiring mentor, and a great collaborator.

## Data Availability

Data sharing not applicable–no new data generated.
